# Effectiveness of Pfizer-BioNTech mRNA Vaccination Against COVID-19 Hospitalization Among Persons Aged 12–18 Years — United States, June–September 2021

**DOI:** 10.15585/mmwr.mm7042e1

**Published:** 2021-10-22

**Authors:** Samantha M. Olson, Margaret M. Newhams, Natasha B. Halasa, Ashley M. Price, Julie A. Boom, Leila C. Sahni, Katherine Irby, Tracie C. Walker, Stephanie P. Schwartz, Pia S. Pannaraj, Aline B. Maddux, Tamara T. Bradford, Ryan A. Nofziger, Benjamin J. Boutselis, Melissa L. Cullimore, Elizabeth H. Mack, Jennifer E. Schuster, Shira J. Gertz, Natalie Z. Cvijanovich, Michele Kong, Melissa A. Cameron, Mary A. Staat, Emily R. Levy, Brandon M. Chatani, Kathleen Chiotos, Laura D. Zambrano, Angela P. Campbell, Manish M. Patel, Adrienne G. Randolph, Overcoming COVID-19 Investigators, Overcoming COVID-19 Investigators, Meghan Murdock, Mary Glas Gaspers, Katri V. Typpo, Connor P. Kelley, Ronald C. Sanders, Masson Yates, Chelsea Smith, Katheryn Crane, Geraldina Lionetti, Juliana Murcia-Montoya, Matt S. Zinter, Denise Villarreal-Chico, Adam L. Skura, Daniel Hakimi, Harvey Peralta, Emily Port, Imogene A. Carson, Justin M. Lockwood, Satoshi Kamidani, Keiko M. Tarquinio, Caitlen E. Taylor, Kelly N. Michelson, Bria M. Coates, Marla S. Johnston, Suden Kucukak, Sabrina R. Chen, Amber O. Orzel, Edie Weller, Laura Berbert, Jie He, Sabrina M. Heidemann, Janet R. Hume, Ellen R. Bruno, Lexie A. Goertzen, Supriya Behl, Noelle M. Drapeau, Shannon M. Hill, Abigail Kietzman, Valerie Rinehart, Lauren A. Hoody, Angelo G. Navas, Paris C. Bennett, Nicole A. Twinem, Merry L. Tomcany, Chelsea C. Rohlfs, Rebecca L. Douglas, Megan M. Bickford, Lauren E. Wakefield, Janet B. Nicotera, Meenakshi Golchha, Jennifer N. Oates

**Affiliations:** ^1^CDC COVID-19 Response Team; ^2^Department of Anesthesiology, Critical Care, and Pain Medicine, Boston Children’s Hospital, Boston, Massachusetts; ^3^Division of Pediatric Infectious Diseases, Department of Pediatrics, Vanderbilt University Medical Center, Nashville, Tennessee; ^4^Department of Pediatrics, Baylor College of Medicine, Immunization Project, Texas Children’s Hospital, Houston, Texas; ^5^Section of Pediatric Critical Care, Department of Pediatrics, Arkansas Children's Hospital, Little Rock, Arkansas; ^6^Department of Pediatrics, University of North Carolina at Chapel Hill Children's Hospital, Chapel Hill, North Carolina; ^7^Division of Infectious Diseases, Children’s Hospital Los Angeles and Departments of Pediatrics and Molecular Microbiology and Immunology, University of Southern California, Los Angeles, California; ^8^Department of Pediatrics, Section of Critical Care Medicine, University of Colorado School of Medicine and Children's Hospital Colorado, Aurora, Colorado; ^9^Department of Pediatrics, Division of Cardiology, Louisiana State University Health Sciences Center and Children’s Hospital of New Orleans, New Orleans, Louisiana; ^10^Division of Critical Care Medicine, Department of Pediatrics, Akron Children’s Hospital, Akron, Ohio; ^11^Division of Pediatric Critical Care, Department of Pediatrics, Children’s Hospital and Medical Center, Omaha, Nebraska; ^12^Division of Pediatric Critical Care Medicine, Medical University of South Carolina, Charleston, South Carolina; ^13^Division of Pediatric Infectious Diseases, Department of Pediatrics, Children’s Mercy Kansas City, Kansas City, Missouri; ^14^Division of Pediatric Critical Care, Department of Pediatrics, Saint Barnabas Medical Center, Livingston, New Jersey; ^15^Division of Critical Care Medicine, University of California, San Francisco Benioff Children's Hospital Oakland, Oakland, California; ^16^Division of Pediatric Critical Care Medicine, Department of Pediatrics, University of Alabama at Birmingham, Birmingham, Alabama; ^17^Division of Pediatric Hospital Medicine, University of California San Diego-Rady Children’s Hospital, San Diego, California; ^18^Department of Pediatrics, University of Cincinnati College of Medicine, Cincinnati Children's Hospital Medical Center, Cincinnati, Ohio; ^19^Divisions of Pediatric Infectious Diseases and Pediatric Critical Care Medicine, Department of Pediatric and Adolescent Medicine, Mayo Clinic, Rochester, Minnesota; ^20^Division of Pediatric Infectious Diseases, Department of Pediatrics, UHealth/Holtz Children's Hospital, Miami, Florida; ^21^Division of Critical Care Medicine, Department of Anesthesiology and Critical Care, Children’s Hospital of Philadelphia, Philadelphia, Pennsylvania; ^22^Departments of Anesthesia and Pediatrics, Harvard Medical School, Boston, Massachusetts.; Children’s of Alabama, Birmingham, Alabama; University of Arizona, Tucson, Arizona; University of Arizona, Tucson, Arizona; University of Arizona, Tucson, Arizona; Arkansas Children’s Hospital, Little Rock, Arkansas; Arkansas Children’s Hospital, Little Rock, Arkansas; Arkansas Children’s Hospital, Little Rock, Arkansas; Rady Children’s Hospital, San Diego, California; University of California, San Francisco Benioff Children’s Hospital Oakland, Oakland, California; University of California, San Francisco Benioff Children’s Hospital Oakland, Oakland, California; University of California, San Francisco Benioff Children’s Hospital, San Francisco, California; University of California, San Francisco Benioff Children’s Hospital, San Francisco, California; Children’s Hospital Los Angeles, Los Angeles, California; Children’s Hospital Los Angeles, Los Angeles, California; Children’s Hospital Los Angeles, Los Angeles, California; Children’s Hospital Colorado, Aurora, Colorado; Children’s Hospital Colorado, Aurora, Colorado; Children’s Hospital Colorado, Aurora, Colorado; Emory University School of Medicine, Children's Healthcare of Atlanta, Atlanta, Georgia; Emory University School of Medicine, Children's Healthcare of Atlanta, Atlanta, Georgia; Emory University School of Medicine, Children's Healthcare of Atlanta, Atlanta, Georgia; Ann & Robert H. Lurie Children’s Hospital of Chicago, Chicago, Illinois; Ann & Robert H. Lurie Children’s Hospital of Chicago, Chicago, Illinois; Children's Hospital of New Orleans, New Orleans, Louisiana; Boston Children’s Hospital, Boston, Massachusetts; Boston Children’s Hospital, Boston, Massachusetts; Boston Children’s Hospital, Boston, Massachusetts; Boston Children’s Hospital, Boston, Massachusetts; Boston Children’s Hospital, Boston, Massachusetts; Boston Children’s Hospital, Boston, Massachusetts; Children’s Hospital of Michigan, Detroit, Michigan; University of Minnesota Masonic Children’s Hospital, Minneapolis, Minnesota; University of Minnesota Masonic Children’s Hospital, Minneapolis, Minnesota; University of Minnesota Masonic Children’s Hospital, Minneapolis, Minnesota; Mayo Clinic, Rochester, Minnesota; Mayo Clinic, Rochester, Minnesota; Children’s Mercy Hospital, Kansas City, Missouri; Children’s Mercy Hospital, Kansas City, Missouri; Children’s Hospital & Medical Center, Omaha, Nebraska; Children’s Hospital & Medical Center, Omaha, Nebraska; University of North Carolina at Chapel Hill, Chapel Hill, North Carolina; University of North Carolina at Chapel Hill, Chapel Hill, North Carolina; Rebecca D. Considine Research Institute, Akron Children’s Hospital; Akron, Ohio; Rebecca D. Considine Research Institute, Akron Children’s Hospital; Akron, Ohio; Cincinnati Children’s Hospital, Cincinnati, Ohio; Children’s Hospital of Philadelphia, Philadelphia, Pennsylvania; Medical University of South Carolina Children’s Health, Charleston, South Carolina; Medical University of South Carolina Children’s Health, Charleston, South Carolina; Monroe Carell Jr. Children’s Hospital at Vanderbilt, Nashville, Tennessee; Monroe Carell Jr. Children’s Hospital at Vanderbilt, Nashville, Tennessee; Texas Children’s Hospital, Houston, Texas

Pfizer-BioNTech COVID-19 vaccine is authorized for use in children and adolescents aged 12–15 years and is licensed by the Food and Drug Administration (FDA) for persons aged ≥16 (*1*). A randomized placebo-controlled trial demonstrated an efficacy of 100% (95% confidence interval [CI] = 75.3%–100%) in preventing outpatient COVID-19 in persons aged 12–15 years (*2*); however, data among adolescents on vaccine effectiveness (VE) against COVID-19 in real-world settings are limited, especially among hospitalized patients. In early September 2021, U.S. pediatric COVID-19 hospitalizations reached the highest level during the pandemic (*3*,*4*). In a test-negative, case-control study at 19 pediatric hospitals in 16 states during June 1–September 30, 2021, the effectiveness of 2 doses of Pfizer-BioNTech vaccine against COVID-19 hospitalization was assessed among children and adolescents aged 12–18 years. Among 464 hospitalized persons aged 12–18 years (179 case-patients and 285 controls), the median age was 15 years, 72% had at least one underlying condition, including obesity, and 68% attended in-person school. Effectiveness of 2 doses of Pfizer-BioNTech vaccine against COVID-19 hospitalization was 93% (95% CI = 83%–97%), during the period when B.1.617.2 (Delta) was the predominant variant. This evaluation demonstrated that 2 doses of Pfizer-BioNTech vaccine are highly effective at preventing COVID-19 hospitalization among persons aged 12–18 years and reinforces the importance of vaccination to protect U.S. youths against severe COVID-19.

This study used a test-negative design, similar to other postauthorization VE evaluations, in which vaccine performance is assessed by comparing the odds of antecedent vaccination among laboratory-confirmed case-patients hospitalized with COVID-19 and hospitalized controls without COVID-19 (*5*). Participants were aged 12–18 years and were admitted to 19 pediatric hospitals in the CDC-funded Overcoming COVID-19 Network during June 1–September 30, 2021 (*6*). Case-patients^†^ were hospitalized with symptomatic COVID-19–like illness and a positive SARS-CoV-2 reverse transcription–polymerase chain reaction (RT-PCR) or antigen test result; no case-patients received a diagnosis of multisystem inflammatory syndrome in children (MIS-C) during their enrolling hospitalization. Two hospitalized control groups were enrolled: 1) patients with symptoms compatible with COVID-19 with negative SARS-CoV-2 RT-PCR or antigen test results (test-negative) and 2) patients without COVID-19–associated symptoms who might or might not have received SARS-CoV-2 testing (syndrome-negative).^§^ Baseline demographic characteristics, clinical information about the current illness, and SARS-CoV-2 testing history were obtained through parent or guardian interviews performed by trained study personnel and review of electronic medical records. Parents or guardians were asked about COVID-19 vaccination history, including number of doses and whether the most recent dose occurred in the last 14 days, location where vaccination occurred, vaccine manufacturer, and availability of a COVID-19 vaccination card. Study personnel searched sources, including state vaccination registries, electronic medical records, or other sources (including documentation from pediatricians) to verify reported or unknown vaccination status.

Patients were considered to have received COVID-19 vaccination based on source documentation or by plausible self-report (vaccination dates and location were provided). Because vaccination with Moderna or Janssen vaccine were not authorized for persons aged <18 years at the time of this evaluation, only receipt of Pfizer-BioNTech vaccine was assessed in this analysis. The study included fully vaccinated persons aged 12–18 years with COVID-19 vaccination status categorized as 1) unvaccinated (no receipt of any COVID-19 vaccine before illness onset^¶^) or 2) fully vaccinated (receipt of 2 doses of Pfizer-BioNTech vaccine, with the second dose administered ≥14 days before illness onset). Patients who were partially vaccinated (i.e., received only 1 dose or received a second dose <14 days before illness onset) were excluded from the analysis. Descriptive statistics were used to compare characteristics of case-patients and controls. Pearson chi-square tests (categorical outcomes) or Wilcoxon rank-sum test for medians (continuous outcomes) were used to make comparisons between groups; statistical significance was defined as p<0.05. VE against COVID-19 hospitalization was calculated by comparing the odds of full COVID-19 vaccination among case-patients and controls using the equation VE = 100 × (1 – adjusted odds ratio), determined from logistic regression models. Firth penalized regression was used for models with six or fewer vaccinated case-patients. Models were adjusted for U.S. Census region, calendar month of admission, age, sex, and race/ethnicity (*5*). Other factors were assessed (underlying health conditions and social vulnerability index) but were not included in the final model because they did not change the odds ratio of vaccination by >5% (*5*). Sensitivity analyses were performed to evaluate whether VE differed by control group. VE was also stratified by age groups (12–15 and 16–18 years). Statistical analyses were conducted using SAS (version 9.4; SAS Institute). This activity was reviewed by CDC and the other participating institutions and was conducted consistent with applicable federal law and CDC policy.**

During June 1–September 30, 2021, among 572 eligible patients, 108 were excluded, including 56 who were partially vaccinated or who completed vaccination 0–13 days before illness onset, 20 who were hospitalized >14 days after illness onset, 14 case-patients who received a positive SARS-CoV-2 test result but were admitted for non–COVID-19 reasons, and 18 who were excluded for other reasons.^††^ The 464 patients in the final analysis comprised 179 case-patients and 285 controls (122 [43%] test-negative and 163 [57%] syndrome-negative). Among case-patients and all controls, the median age was 15 years, 72% had at least one underlying condition, including obesity, and 68% attended in-person school (Table 1). Vaccination coverage was 3% among case-patients and 33% among controls. Case-patients more frequently resided in areas with higher social vulnerability index scores^§§^ (median = 0.67) than did controls (median = 0.58) (p = 0.02). The distribution of most underlying conditions was not significantly different between case-patients and controls; however, diabetes was more prevalent among case-patients (12%) than among controls (5%) (p = 0.01), and neurologic or neuromuscular disorders were more prevalent among controls (28%) than among case-patients (12%) (p<0.01).

**TABLE 1:** Characteristics of hospitalized COVID-19 case-patients and controls aged 12–18 years — 19 pediatric hospitals, 16 states,* June–September 2021

Characteristic (no. unknown)	Case status, no. (column %)			P-value^†^
	Total (N = 464)	Case-patients (n = 179)	Controls (n = 285)	
**Median age, yrs (IQR)**	15 (14–17)	16 (14–17)	15 (14–17)	0.07
**Age group, yrs**				
12–15	285 (61.4)	106 (59.2)	179 (62.8)	0.44
16–18	179 (38.6)	73 (40.8)	106 (37.2)	
**Sex**				
Female	210 (45.3)	90 (50.3)	120 (42.1)	0.09
**Race/Ethnicity**				
White, non-Hispanic	193 (41.6)	68 (38.0)	125 (43.9)	0.27
Black, non-Hispanic	96 (20.7)	37 (20.7)	59 (20.7)	
Hispanic, any race	125 (26.9)	57 (31.8)	68 (23.9)	
Other, non-Hispanic	33 (7.1)	13 (7.3)	20 (7.0)	
Unknown	17 (3.7)	4 (2.2)	13 (4.6)	
**Social vulnerability index,**^§^ **median (IQR) (1)**	0.60 (0.34–0.82)	0.67 (0.37–0.85)	0.58 (0.32–0.80)	0.02
U.S. Census region*				
Northeast	21 (4.5)	5 (2.8)	16 (5.6)	0.28
Midwest	60 (12.9)	28 (15.6)	32 (11.2)	
South	283 (61.0)	106 (59.2)	177 (62.1)	
West	100 (21.6)	40 (22.4)	60 (21.1)	
**Month of admission**				
June	21 (4.5)	7 (3.9)	14 (4.9)	0.03
July	50 (10.8)	29 (16.2)	21 (7.4)	
August	159 (34.3)	58 (32.4)	101 (35.4)	
September	234 (50.4)	85 (47.5)	149 (52.3)	
**Underlying health condition**				
At least one underlying condition (2)	333 (72.1)	131 (73.2)	202 (71.4)	0.67
Respiratory system disorder (4)	120 (26.1)	55 (30.9)	65 (23.1)	0.06
Asthma (6)	88 (19.2)	42 (23.7)	46 (16.4)	0.05
Cardiovascular system disorder (5)	29 (6.3)	7 (3.9)	22 (7.8)	0.09
Neurologic/Neuromuscular disorder (3)	100 (21.7)	21 (11.8)	79 (27.9)	<0.01
Active or prior oncologic disorder (3)	25 (5.4)	6 (3.4)	19 (6.7)	0.12
Nononcologic immunosuppressive disorder (5)	9 (2.0)	2 (1.1)	7 (2.5)	0.31
Endocrine disorder (3)	63 (13.7)	30 (16.8)	33 (11.7)	0.12
Diabetes (4)	35 (7.6)	21 (11.8)	14 (5.0)	0.01
Other chronic conditions^¶^ (2)	226 (48.9)	100 (55.9)	126 (44.5)	0.02
**Other characteristic**				
In-person school attendance (161)	205 (67.7)	80 (68.4)	125 (67.2)	0.83
Fully vaccinated**	99 (21.3)	6 (3.4)	93 (32.6)	<0.01
If fully vaccinated, median days from second vaccine to illness onset (IQR)**^††^**	72 (45–97)	55 (47–106)	73 (43–97)	0.68

**Abbreviations:** IQR = interquartile range; SVI = social vulnerability index.

* Patients were enrolled from 19 pediatric hospitals in 16 states. *Northeast*: Boston Children's Hospital (Massachusetts), Saint Barnabas Medical Center (New Jersey), *Midwest*: Akron Children’s Hospital (Ohio), Children’s Mercy Kansas City (Missouri), Children’s Hospital and Medical Center: Nebraska (Nebraska), Cincinnati Children’s Hospital Medical Center (Ohio), Mayo Clinic (Minnesota), *South*: Arkansas Children's Hospital (Arkansas), University of North Carolina at Chapel Hill Children’s Hospital (North Carolina), Children’s of Alabama (Alabama), Monroe Carell Jr. Children's Hospital at Vanderbilt (Tennessee), Medical University of South Carolina Children’s Health (South Carolina), Texas Children’s Hospital (Texas), Holtz Children’s Hospital (Florida), Children’s Hospital of New Orleans (Louisiana), *West*: University of California San Francisco Benioff Children’s Hospital Oakland (California), Children's Hospital Colorado (Colorado), Children’s Hospital Los Angeles (California), University of California San Diego-Rady Children’s Hospital (California).

^†^ Testing for statistical significance was conducted using the Pearson chi-square test to compare categorical variables or Wilcoxon rank-sum test for medians to compare continuous data.

^§^ CDC/ATSDR SVI documentation is available at https://www.atsdr.cdc.gov/placeandhealth/svi/index.html. Median SVI for case-patients and controls are based on US 2018 SVI data.

^¶^ Other chronic conditions included rheumatologic/autoimmune disorder, hematologic disorder, renal or urologic dysfunction, gastrointestinal/hepatic disorder, metabolic or confirmed or suspected genetic disorder (including obesity), or atopic or allergic condition.

** COVID-19 vaccination status included the following two categories: 1) unvaccinated, defined as no receipt of any SARS-CoV-2 vaccine before illness onset and 2) fully vaccinated, defined as receipt of both doses of a 2-dose Pfizer-BioNTech vaccination ≥14 days before illness onset.

^††^ Dates are based on those with documented vaccination, not plausible self-report. The date of illness onset was used for case-patients and controls with COVID-19–like illness with median value imputed if missing. For controls without COVID-19–like illness, the date of admission was used for a date of illness onset, also referred to as illness onset for this report.

Among 179 COVID-19 case-patients, six (3%) were vaccinated and 173 (97%) were unvaccinated (Table 2). Overall, 77 (43%) case-patients were admitted to an intensive care unit, and 29 (16%) critically ill case-patients received life support during hospitalization, including invasive mechanical ventilation, vasoactive infusions, or extracorporeal membrane oxygenation; two of these 29 critically ill patients (7%) died. All 77 case-patients admitted to the intensive care unit, all 29 critically ill case-patients, and both deaths occurred among unvaccinated case-patients. Among 169 case-patients with available hospital discharge data, the median length of hospital stay was 5 days (interquartile range [IQR] = 2–9 days) for unvaccinated case-patients and 3 days (IQR = 2–4 days) for vaccinated case-patients.

**TABLE 2:** Clinical outcomes and severity among hospitalized COVID-19 case-patients aged 12–18 years, by vaccination status* — 19 pediatric hospitals, 16 states,^†^ June–September 2021

Characteristic (no. unknown)	Case-patients hospitalized with COVID-19, no. (%)		
	Total (N = 179)	Unvaccinated (n = 173)	Fully vaccinated (n = 6)
**ICU admission**	**77 (43.0)**	**77 (44.5)**	**0 (—)**
**Critically ill patients on life support**	**29 (16.2)**	**29 (16.8)**	**0 (—)**
Invasive mechanical ventilation	21 (11.7)	21 (12.1)	0 (—)
Vasoactive infusions (1)	20 (11.2)	20 (11.6)	0 (—)
Extracorporeal membrane oxygenation (2)	7 (4.0)	7 (4.1)	0 (—)
**Patients with discharge data, no./total no (%)**	**172/179 (96.1)**	**166/173 (96.0)**	**6/6 (100)**
Hospital length of stay, median (IQR) (10)	5 (2–9)	5 (2–9)	3 (2–4)
Died before discharge (7)	2 (1.2)	2 (1.2)	0 (—)

**Abbreviations:** ICU = intensive care unit; IQR = interquartile range.

* COVID-19 vaccination status included the following two categories: 1) unvaccinated, defined as no receipt of any SARS-CoV-2 vaccine before illness onset and 2) fully vaccinated, defined as receipt of both doses of a 2-dose Pfizer-BioNTech vaccination ≥14 days before illness onset.

^†^ Patients were vaccinated and unvaccinated persons aged 12–18 years enrolled from 19 pediatric hospitals in 16 states. *Northeast*: Boston Children's Hospital (Massachusetts), Saint Barnabas Medical Center (New Jersey), *Midwest*: Akron Children’s Hospital (Ohio), Children’s Mercy Kansas City (Missouri), Children’s Hospital and Medical Center: Nebraska (Nebraska), Cincinnati Children’s Hospital Medical Center (Ohio), Mayo Clinic (Minnesota), *South*: Arkansas Children's Hospital (Arkansas), University of North Carolina at Chapel Hill Children’s Hospital (North Carolina), Children’s of Alabama (Alabama), Monroe Carell Jr. Children's Hospital at Vanderbilt (Tennessee), Medical University of South Carolina Children’s Health (South Carolina), Texas Children’s Hospital (Texas), Holtz Children’s Hospital (Florida), Children’s Hospital of New Orleans (Louisiana), *West*: University of California San Francisco Benioff Children’s Hospital Oakland (California), Children's Hospital Colorado (Colorado), Children’s Hospital Los Angeles (California), University of California San Diego-Rady Children’s Hospital (California).

VE against COVID-19 hospitalization was 93% (95% CI = 83%–97%) (Table 3), during the period when B.1.617.2 (Delta) was the predominant variant. Among all 99 patients classified as fully vaccinated, 96 (97%) had documentation of vaccination status. In a sensitivity analysis, VE was similar for each control group assessed independently (test-negative VE = 94%, 95% CI = 85%–98%; syndrome-negative VE = 92%, 95% CI = 80%–97%). In addition, VE was similar among 106 case-patients aged 12–15 years (VE = 91%) and 73 case-patients aged 16–18 years (VE = 94%).

**TABLE 3:** Vaccine effectiveness* against COVID-19 among hospitalized patients aged 12–18 years, by vaccination status^†^ — 19 pediatric hospitals, 16 states,^§^ July–September 2021

Age group, yrs	No. vaccinated/Total (%)		Vaccine effectiveness, % (95% CI)
	Case-patients	Controls	
**All**	**6/179 (3.4)**	**93/285 (32.6)**	**93 (83–97)**
12–15	4/106 (3.8)	53/179 (29.6)	91 (74–97)
16–18	2/73 (2.7)	40/106 (37.7)	94 (78–99)

**Abbreviation:** CI = confidence interval.

* Vaccine effectiveness estimates were based on odds of antecedent vaccination in case-patients vs controls adjusted for U.S. Census region, calendar month of admission, continuous age in years, sex, race/ethnicity (non-Hispanic White, non-Hispanic Black, non-Hispanic other, Hispanic of any race, or unknown). Firth penalized regression was used for models with six or fewer vaccinated cases.

^†^ COVID-19 vaccination status included the following two categories: 1) unvaccinated, defined as no receipt of any SARS-CoV-2 vaccine before illness onset and 2) fully vaccinated, defined as receipt of both doses of a 2-dose Pfizer-BioNTech vaccination ≥14 days before illness onset.

^§^ Patients were enrolled from 19 pediatric hospitals in 16 states. *Northeast*: Boston Children's Hospital (Massachusetts), Saint Barnabas Medical Center (New Jersey), *Midwest*: Akron Children’s Hospital (Ohio), Children’s Mercy Kansas City (Missouri), Children’s Hospital and Medical Center: Nebraska (Nebraska), Cincinnati Children’s Hospital Medical Center (Ohio), Mayo Clinic (Minnesota), *South*: Arkansas Children's Hospital (Arkansas), University of North Carolina at Chapel Hill Children’s Hospital (North Carolina), Children’s of Alabama (Alabama), Monroe Carell Jr. Children's Hospital at Vanderbilt (Tennessee), Medical University of South Carolina Children’s Health (South Carolina), Texas Children’s Hospital (Texas), Holtz Children’s Hospital (Florida), Children’s Hospital of New Orleans (Louisiana), *West*: University of California San Francisco Benioff Children’s Hospital Oakland (California), Children's Hospital Colorado (Colorado), Children’s Hospital Los Angeles (California), University of California San Diego-Rady Children’s Hospital (California).

## Discussion

During June–September 2021, receipt of 2 doses of Pfizer-BioNTech vaccine provided a high level of protection against COVID-19 hospitalization among children and adolescents aged 12–18 years in a real-world evaluation at 19 U.S. pediatric hospitals. This evaluation demonstrated that nearly all (97%) persons aged 12–18 years hospitalized with COVID-19 were unvaccinated (versus fully vaccinated) and reinforces the importance of vaccination to protect U.S. youths against severe COVID-19.

These findings are consistent with efficacy data from the Pfizer-BioNTech clinical trial among persons aged 12–15 years, which found an observed vaccine efficacy of 100% (95% CI = 75.3%–100%) (*2*). However, that trial was not powered to assess efficacy against hospitalized COVID-19. Another study reported VE against COVID-19 hospitalization of 81% for fully vaccinated patients aged 12–15 years; however, that study assessed only 45 cases and thus had wide CIs (–55% to 98%) (*7*). One other evaluation from Israel evaluated Pfizer-BioNTech VE against SARS-CoV-2 infection in patients aged 12–15 years and found similarly high VE (91.5%; 95% CI = 88.2%–93.9%), but the study did not include enough cases to examine VE against hospitalized COVID-19 (*8*). In this real-world analysis, in which all case-patients were hospitalized, vaccination reduced the risk for COVID-19 hospitalization in persons aged 12–18 years by 93%. Moreover, 16% of patients hospitalized with COVID-19 had critical illness requiring life support; all were unvaccinated. Taken together, these findings contribute to the growing knowledge regarding VE against pediatric COVID-19, as updated FDA Emergency Use Authorizations to expand COVID-19 vaccine eligibility to younger ages are considered.

The findings in this report are subject to at least six limitations. First, VE could not be assessed directly against specific variants; the predominant variant during the evaluation period was B.1.617.2 (Delta) (*9*). Second, the sample was too small to assess VE by underlying conditions or by other subgroups of interest, including against critical illness. Third, because this analysis included self-reported data from some participants, vaccination status might have been misclassified in a few case-patients or controls, or there might have been imperfect recollection of illness onset dates. Fourth, because of high levels of COVID-19 transmission in southern states during this period, the majority of patients in this analysis (61%) were from the South; this might limit the representativeness of the sample. Fifth, this report only assessed VE for the Pfizer-BioNTech vaccine. Finally, because vaccination of persons aged 12–15 years commenced only recently, evaluation of duration of protection was not possible.

As of October 18, 2021, 46% of U.S. children and adolescents aged 12–15 years and 54% of those aged 16–17 years were fully vaccinated against COVID-19 (*10*). In a multistate network of U.S. pediatric hospitals, this study found that receipt of 2 doses of Pfizer-BioNTech vaccine was highly effective in preventing COVID-19 hospitalization among persons aged 12–18 years. These data suggest that increasing vaccination coverage among this group could reduce the incidence of severe COVID-19 in the United States. Further, as in-person school attendance increases, multicomponent preventive measures to reduce the incidence of severe COVID-19 among adolescents, including vaccination, are imperative.^¶¶^

**Summary**

**What is already known about this topic?**

Persons aged 12–18 years are eligible to receive COVID-19 vaccine. Currently, data are lacking on real-world vaccine effectiveness against COVID-19 hospitalization in adolescents.

**What is added by this report?**

Among hospitalized U.S. patients aged 12–18 years, vaccine effectiveness of 2 doses of Pfizer-BioNTech vaccine against COVID-19 hospitalization during June–September 2021, was 93% (95% confidence interval = 83%–97%).

**What are the implications for public health practice?**

This evaluation demonstrated that 2 doses of Pfizer-BioNTech vaccine were highly effective in preventing COVID-19 hospitalization among persons aged 12–18 years. Findings reinforce the importance of vaccination to protect U.S. youths against severe COVID-19.

## Footnotes

* These authors contributed equally to this report.

† Symptomatic COVID-19–like illness was defined as one or more of the following: fever, cough, shortness of breath, loss of taste, loss of smell, gastrointestinal symptoms (e.g., diarrhea, vomiting, or stomachache), use of respiratory support (e.g., high flow oxygen by nasal cannula, new invasive or noninvasive ventilation) for the acute illness, or new pulmonary findings on chest imaging consistent with pneumonia. Patients with COVID-19 as the primary reason for admission were categorized as symptomatic COVID-19 patients. Seventeen case-patients had some missing data on positive testing and were not retested at the hospital: 15 patients had positive test results with a date and unconfirmed test type, and two patients had positive test results but were missing the date of testing.

§ Syndrome-negative controls had no signs or symptoms of COVID-19 (including fever, cough, shortness of breath, loss of taste, loss of smell, gastrointestinal symptoms, use of respiratory support for the acute illness, or new pulmonary findings on chest imaging consistent with pneumonia) and were not clinically suspected to have COVID-19. Among 163 syndrome-negative controls, 10 (6%) did not receive SARS-CoV-2 testing.

¶ The date of illness onset was used for case-patients and controls with COVID-19–like illness with median value imputed if missing. For controls without COVID-19–like illness, the date of admission was used for a date of illness onset, also referred to as illness onset for this report.

** 45 C.F.R. part 46.102(l)(2), 21 C.F.R. part 56; 42 U.S.C. Sect. 241(d); 5 U.S.C. Sect. 552a; 44 U.S.C. Sect. 3501 et seq.

†† Other reasons for excluding patients from the analysis included SARS-CoV-2 testing >10 days after illness onset or >3 days from hospitalization (three), onset of COVID-19–like illness after admission (14), and documentation of full vaccination with Moderna COVID-19 vaccine (one).

§§ Documentation for CDC/ATSDR social vulnerability index (SVI) is available at https://www.atsdr.cdc.gov/placeandhealth/svi/index.html. Median SVI for case-patients and controls are based on US 2018 SVI data.

¶¶ Guidance for COVID-19 prevention in kindergarten through grade 12 schools is available at https://www.cdc.gov/coronavirus/2019-ncov/community/schools-childcare/k-12-guidance.html.
